# Supplementary material for: Two Male-Specific Antimicrobial Peptides SCY2 and Scyreprocin as Crucial Molecules Participated in the Sperm Acrosome Reaction of Mud Crab Scylla paramamosain
Source: Int J Mol Sci. 2022 Mar 21;23(6):3373. doi: 10.3390/ijms23063373 (PMC8952799; doi:10.3390/ijms23063373)
Supplement: Supplementary file 1 [file ijms-23-03373-s001.zip › ijms-1636291-supplementary.pdf]

# Supplementary materials

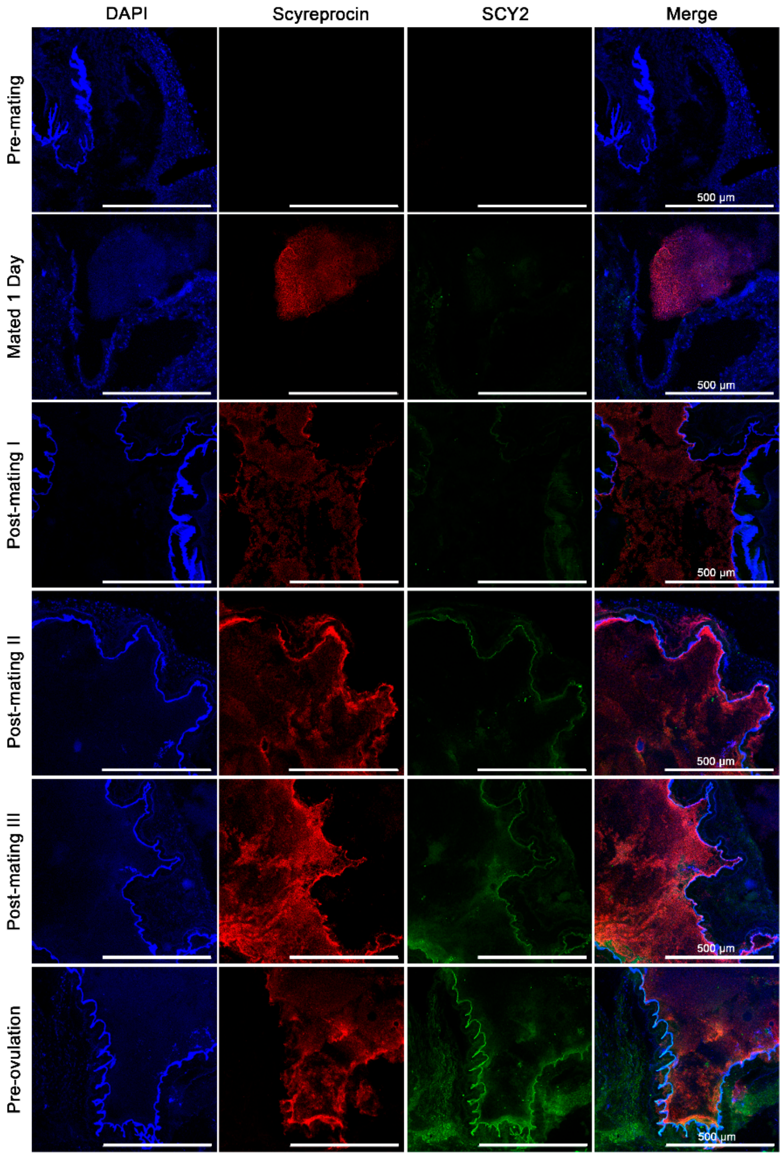


**Figure S1.** Expression patterns of scyreprocin and SCY2 in spermathecae of post-mating female *Scylla paramamosain*.

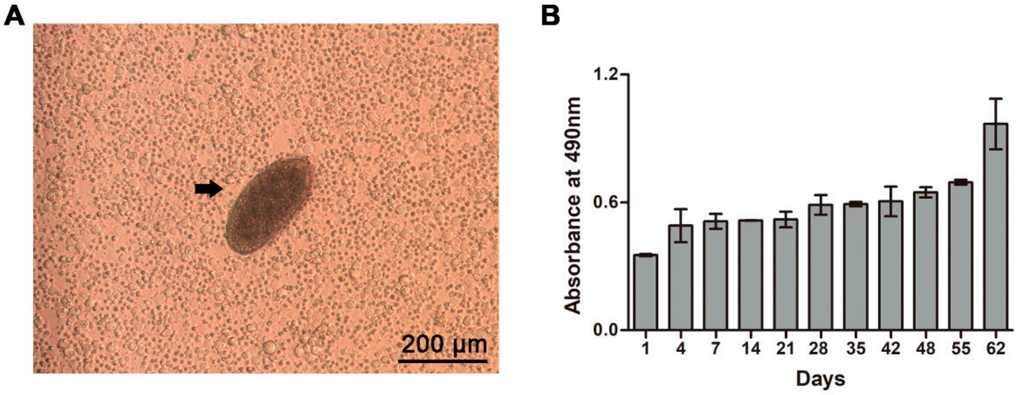


**C**

```
1   TGTACAAGCC CAATTAAGGT GAAACCGCGA ATGGCTCATT AAATCAGCTA TGATTCATTG
61  GATCTGTAGC CCACACTTAC TTGGATAACT GTGGTAATTC TAGAGCTAAT ACATGCATCA
121 CGTCTCTGAC CGCAAGGGAA GAGCGCTTTT ATTAGTTCAA AACCGGTCGG GCCTCGGTCC
181 GCCAACCCCA CCGTGTTGAA TCTGAATAAC TTGTGGCTGA GCGCACGGCC TCCAGTGCCG
241 GCGCCGCCTC TTTCAAGTGT CTGCCTTATC AGCTTTCGAT TGTAGGCTAT GCGCCTACAA
301 TGGCTATAAC GGGTAACGGG GAATCAGGGT TCGATTCCGG AGAGGGAGCC TGAGAAACGG
361 CTACCACATC TAAGGAAGGC AGCAGGCACG CAAATTACCC ACTCCCGGCA CGGGGAGGTA
421 GTGACGAAAA ATAACGATGC GAGACTCATC CGAGGCCTCG CAATCGAAAA TGAGTACA
```

**Figure S2. Primary cultivation of the testicular cells of *Scylla paramamosain*. (A)** Bright-field image of the cultivated testicular cells; black arrow indicates a spermatophore. **(B)** Viability of the cultivated testicular cells (n = 3). Data are presented in mean ± standard deviation (SD). **(C)** Result of 18S rDNA sequencing of the cultivated testicular cells.

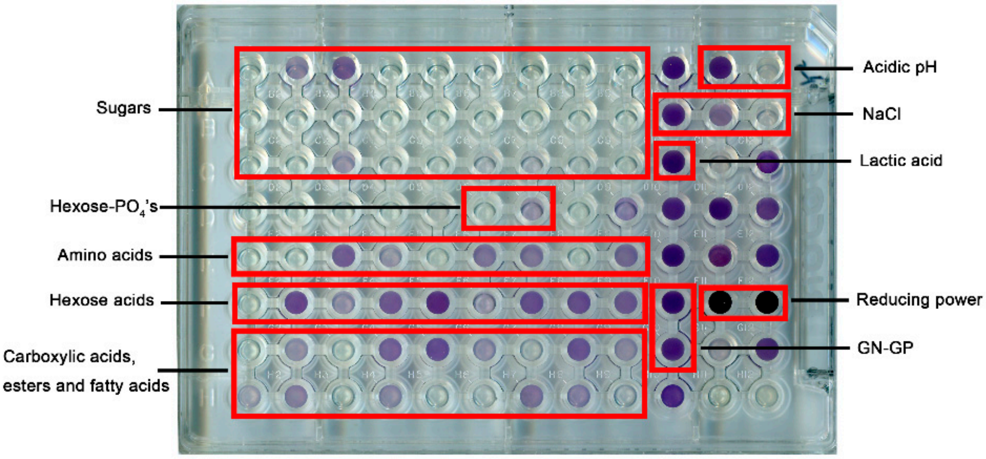


**Well color values**

| Plate | 1 | 2 | 3 | 4 | 5 | 6 | 7 | 8 | 9 | 10 | 11 | 12 |
|---|---|---|---|---|---|---|---|---|---|---|---|---|
| A | 34 | <170- | <299- | 36 | 43 | 52 | 43 | 41 | 39 | <291 | <288 | {188 |
| B | 43 | 41 | 47 | 40 | 43 | 40 | 43 | 41 | 34 | <290 | <255 | {138 |
| C | 52+ | 43 | <169 | 54 | 41 | {74 | {80 | 56 | 43 | <296 | 117+ | {246 |
| D | 44 | 41 | 50 | 42 | {133 | 47 | {117 | 37 | <230 | <281 | <297 | <276 |
| E | 57 | 37 | <249 | <190 | 64+ | <208 | <209 | 37 | <221 | <295 | {247 | <278 |
| F | 62 | <253 | {132 | <247 | <262 | {106 | <256 | <246 | <246 | <295 | <406 | <387 |
| G | 42+ | {158 | 60 | <229 | <254 | {136 | {116 | <245 | <166 | <283 | {175 | <278 |
| H | {153 | <213 | {69 | <170 | {90 | 61 | <209 | <184 | {94 | <272 | 59 | {131 |

Key: <x: positive, x: negative, <x-: mismatched positive, x+:mismatched negative, {x: borderline, -x: less than A1 well

**Average Maxium Positive (Percent)**

| Plate | 1 | 2 | 3 | 4 | 5 | 6 | 7 | 8 | 9 | 10 | 11 | 12 |
|---|---|---|---|---|---|---|---|---|---|---|---|---|
| A | 0 | 0 | 0 | 0 | 0 | 0 | 0 | 0 | 0 | 100 | 100 | 52 |
| B | 0 | 0 | 0 | 0 | 0 | 0 | 0 | 0 | 0 | 97 | 71 | 14 |
| C | 71 | 24 | 52 | 2 | 0 | 17 | 5 | 0 | 8 | 100 | 98 | 84 |
| D | 0 | 0 | 0 | 0 | 78 | 0 | 5 | 0 | 63 | 94 | 98 | 53 |
| E | 0 | 0 | 77 | 80 | 74 | 82 | 82 | 46 | 77 | 98 | 84 | 98 |
| F | 10 | 80 | 39 | 81 | 84 | 62 | 82 | 85 | 81 | 98 | 100 | 100 |
| G | 70 | 14 | 0 | 81 | 84 | 83 | 65 | 82 | 10 | 93 | 34 | 61 |
| H | 17 | 84 | 7 | 60 | 14 | 16 | 46 | 68 | 33 | 48 | 22 | 27 |

**Average Maxium Positive (Percent)**

| Plate | 1 | 2 | 3 | 4 | 5 | 6 | 7 | 8 | 9 | 10 | 11 | 12 |
|---|---|---|---|---|---|---|---|---|---|---|---|---|
| A | ○ | ○ | ○ | ○ | ○ | ○ | ○ | ○ | ○ | ● | ● | ◑ |
| B | ○ | ○ | ○ | ○ | ○ | ○ | ○ | ○ | ○ | ● | ◑ | ○ |
| C | ◑ | ◑ | ◑ | ○ | ○ | ○ | ○ | ○ | ○ | ● | ● | ● |
| D | ○ | ○ | ○ | ○ | ◑ | ○ | ○ | ○ | ◑ | ● | ● | ◑ |
| E | ○ | ○ | ◑ | ● | ◑ | ● | ● | ◑ | ◑ | ● | ● | ● |
| F | ○ | ● | ◑ | ● | ● | ◑ | ● | ● | ● | ● | ● | ● |
| G | ◑ | ○ | ○ | ● | ● | ● | ◑ | ● | ○ | ● | ◑ | ◑ |
| H | ○ | ● | ○ | ◑ | ○ | ○ | ◑ | ◑ | ◑ | ◑ | ◑ | ◑ |

**Result output**

| Rank | PROB | SIM | DIST | Organism Type | Species |
|---|---|---|---|---|---|
| 1 | 0.596 | 0.596 | 5.867 | GN-Nent | *Pseudomonas putida* |
| 2 | 0.135 | 0.135 | 6.522 | GN-Nent | *Pseudomonas mendocina* |
| 3 | 0.029 | 0.029 | 7.437 | GN-Nent | *Pseudomonas viridilivida* |
| 4 | 0.012 | 0.012 | 7.987 | GN-Nent | *Pseudomonas syringae pv maculicola* |

**Figure S3.** Identification of the isolated endogenous microbe by BIOLOG.

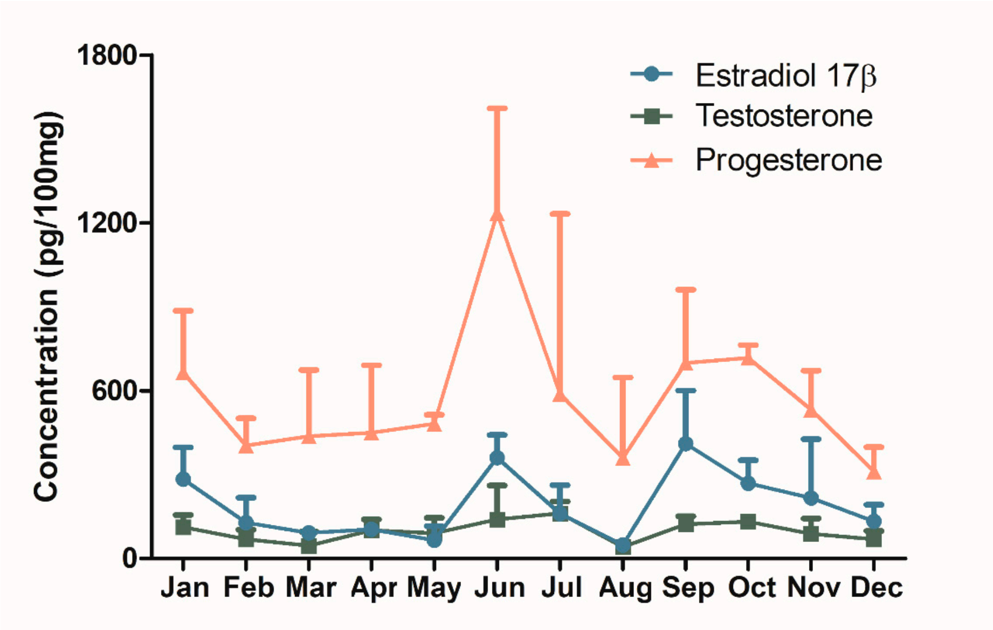


**Figure S4.** Hormone levels in ejaculatory ducts of adult male crabs in different months of the year. Data are presented as mean ± standard deviation (SD) (n = 3).

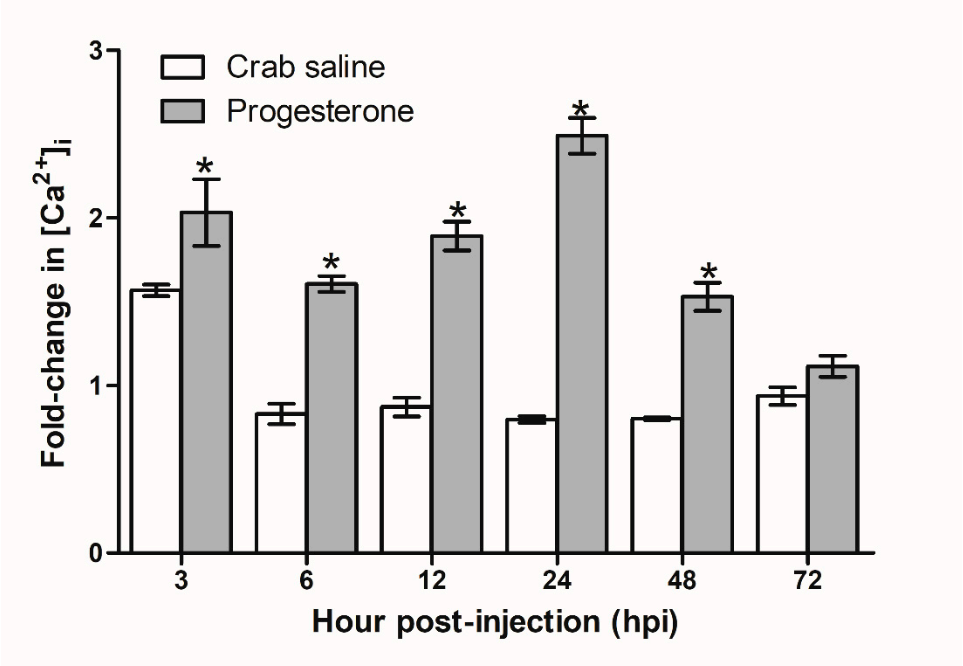


**Figure S5. Changes of intracellular $Ca^{2+}$ concentration in sperm collected from male crabs after progesterone challenge.** Data are presented as mean ± standard deviation (SD) (n = 3). **P* < 0.05, two-way analysis of variance (ANOVA) and Bonferroni post test.

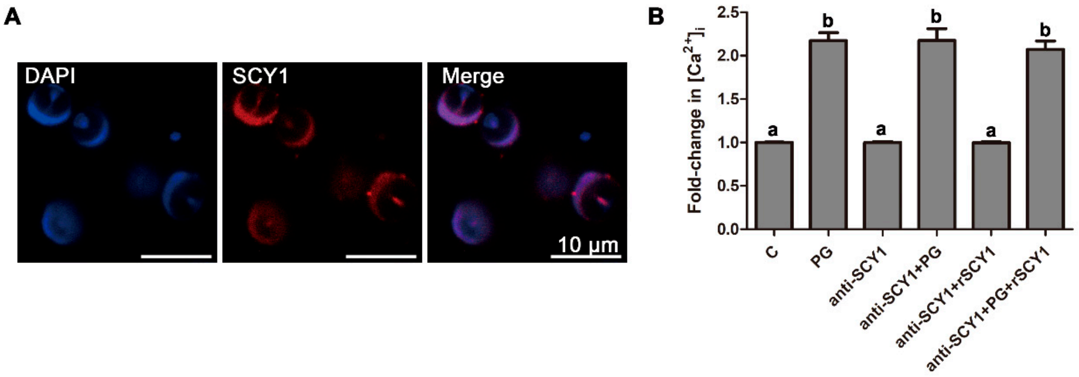


**Figure S6. Scygonadin (SCY1) showed no involvement in the progesterone (PG)-induced sperm acrosome reaction (AR). (A)** Subcellular location of SCY1 in sperm. Scale bars: 10 μm. **(B)** Evaluation of intracellular $Ca^{2+}$ concentration ($[Ca^{2+}]_i$) in sperm samples with different treatments (n = 3). Data are presented as mean ± standard deviation (SD). Letters denote significant differences, one-way analysis of variance (ANOVA) and Tukey post-test.

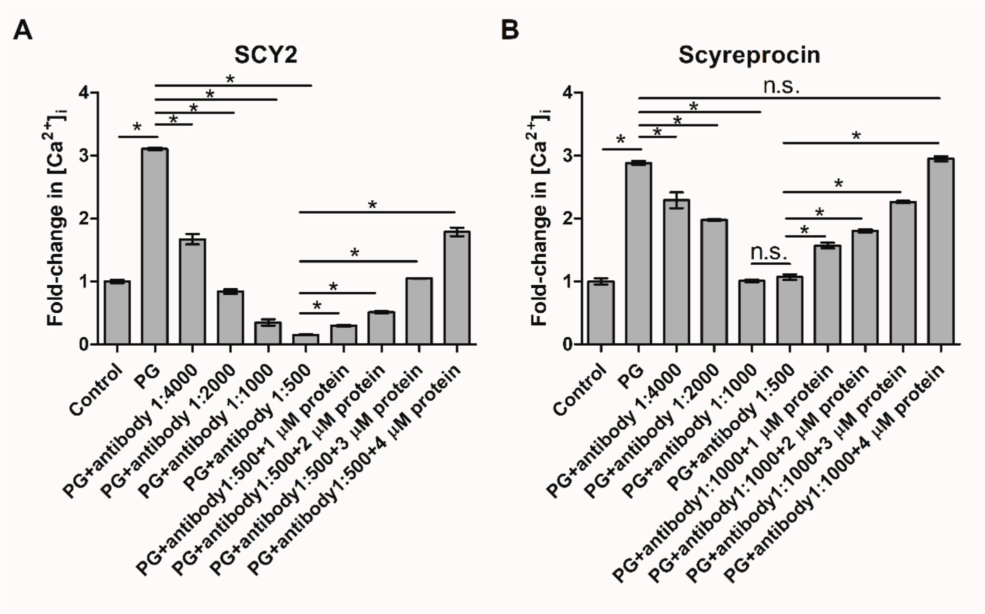


**Figure S7.** Optimization of the antibodies and supplemented recombinant proteins applied in sperm acrosome reaction-induction assays. (A) Optimization of SCY2 antibody dosage (n = 3). (B) Optimization of scyreprocin antibody dosage (n = 3). Data are presented as mean ± standard deviation (SD), $^*P < 0.05$, one-way analysis of variance (ANOVA) and Tukey post-test; n.s., not significant.

**Table S1.** Primer sequences.

| Primer name | Forward Primer | Reverse Primer |
|---|---|---|
| | ***For qRT-PCR*** | |
| qSp-β-actin (GU992421) | GCCCTTCCTCACGCTATCCT | GCGGCAGTGGTCATCTCCT |
| qScyreprocin (MH488960) | GAACAAGGCCCTGCTCTTCACT | TTGCCACTGGGTGAGGGGATA |
| | ***For primary testicular cell culture identification*** | |
| Sp18S (FJ774906) | GTACAAGCCCAATTAAGGTGAAAC | GTACTCATTCCGATTGCGAGGCCT |
| | ***For bacterial identification*** | |
| 16S | AGAGTTTGATCMTGGCTCAG[a] | GGTTACCTTGTTACGACTT[b] |

[a]Universal primer sequence for bacterial 16S rDNA, 27F;

[b]Universal primer sequence for bacterial 16S rDNA, 1492R.

**Text S1. Supplementary Materials and Methods**

**Primary testicular cell culture.**

The culture medium formulation for primary testicular cells was based on a previous study with the slight modification: supplied Leibovitz L-15 with 430 mM NaCl, 11.3 mM KCl, 25 mM $MgCl_2$, 13.3 mM $CaCl_2$, 23 mM $Na_2SO_4$ and 10 mM HEPES, pH adjusted to 7.4 and sterilized by filtration (0.22 μm) [1]. An additional L-glucose (1 g $mL^{-1}$) was freshly supplemented before use. Aseptically excised testes were cut into small pieces in culture medium, dissociated by repeatedly passing through a 1-mL pipette, and filtered through a cell strainer (100 nm) to remove cell clumps. Cell suspension was then seeded at $5\times10^5$ cells $well^{-1}$. The cell proliferation assay was carried out in triplicate using CellTiter 96® AQueous (Promega, Madison, WA). Cell morphology was observed by a Zeiss optical microscopy. The DNA of cultured testicular cells was extracted using TIANamp Marine Animals DNA Kit (TIANGEN Biotech Co. Ltd., Beijing, CHN) and used in 18S rDNA amplification (primer sequences were listed in Table S1) and sequencing.

**Identification of endogenous bacteria.**

Testicular cells with endogenous bacteria proliferation were plated on Difco® marine broth 2216E agar plates and incubated overnight at 28°C. Endogenous bacteria was purified by the streak plate method. The variable regions of 16S rDNA were cloned, sequenced (primer sequences listed in S1 Table) and analyzed using the NCBI/BLAST database. Isolated bacterial strain was identified using the Biolog's identification system (GEN III database, version 5.2.1, Biolog Inc., Hayward, CA) following the manufacturer's instruction.

**Scygonadin protein expression, purification and preparation of polyclonal antibody.**

Scygonadin (SCY1) was expressed and purified as described before [2]. Anti-SCY1 antibody was prepared as mentioned in prior study [3].

**Optimization of the SCY2 and/or scyreprocin-inhibition method.**

Sperm were suspended (~$1\times10^6$ cells $mL^{-1}$) in $Ca^{2+}$-FASW containing different dilutions (1:500-1:4,000) of anti-SCY2 antibody and anti-scyreprocin antibody, respectively (sperm suspended in $Ca^{2+}$-FASW was set as control). Samples were incubated at 28 °C with gentle rotation for 8 h. Sperm cells were harvested by centrifugation (300 g, 10 min), washed with $Ca^{2+}$-FASW, and resuspended in ASW (containing 0.3% (w/v) $Ca^{2+}$) supplied with 50 μg $mL^{-1}$ PG. After a 24 h-incubation, each sample were submitted to $[Ca^{2+}]_i$ measurement. Samples showed an optimized dilution factor of SCY2 (1:500) and scyreprocin (1:1,000) were then supplied with different final concentrations (1-4 μM) of rSCY2 and rScyreprocin, respectively, and incubated for another 1 h before $[Ca^{2+}]_i$ evaluation. The experiment was performed in triplicate.

**Evaluation of hormone levels.**

Every month of the year 2012, ejaculatory ducts (EDs) from 3 crabs (300 ± 20 g) were assayed for PG, testosterone and estradiol levels using ELISA kits (Cayman Chemical Company, Ann Arbor, MI). Tissues (~50 mg) were ground in liquid nitrogen and mixed with 1 mL of ELISA buffer (Cayman Chemical Company); supernatants were subjected to ELISA following manufacturer's instructions. Analyses were carried out in duplicate.

**Evaluation of intracellular $Ca^{2+}$ concentration of sperm after *in vivo* progesterone treatment.**

Male crabs (250 ± 10 g) were injected with 0.1 μg PG $g^{-1}$ body weight (crab saline injection as control), genital ducts were collected in $Ca^{2+}$-FASW (28.05 g NaCl, 4.07 g $MgCl_2\cdot6H_2O$, 7.39 g $MgSO_4\cdot7H_2O$ and 2.38 g HEPES, dissolved in 1 L deionized water and steriled) at 3, 6, 12, 24 and 48 h after injection (n = 3). Sperm were loaded with Fluo-4/AM

probes and measured for fluorescence intensity with a microplate reader (TECAN GENios; Tecan Group Ltd., Männedorf, Switzerland) with Fluo-4/AM excitation at 480 nm and emission at 520 nm.

**Statistical analysis.**

Samples were compared statistically using the IBM SPSS statistics (version 22; IBM Corp., Armonk, NY) and GraphPad Prism software (version 5.01; GraphPad Software Inc., San Diego, CA). One-way analysis of variance (ANOVA) followed by Tukey post tests were applied to analysis changes in $[Ca^{2+}]_i$ of sperm under different treatments. One-way ANOVA and Bonferroni post test were applied to compare the change in $[Ca^{2+}]_i$ of sperm induced by PG-injection. Significant levels were accepted at $^{*}p < 0.05$.

## References


1. Qiao, K.; Xu, W. F.; Chen, H. Y.; Peng, H.; Zhang, Y. Q.; Huang, W. S.; Wang, S. P.; An, Z.; Shan, Z. G.; Chen, F. Y.; Wang, K. J., A new antimicrobial peptide SCY2 identified in *Scylla paramamosain* exerting a potential role of reproductive immunity. *Fish Shellfish Immun* **2016,** 51, 251-262. https://doi.org/10.1016/j.fsi.2016.02.022
2. Peng, H.; Liu, H. P.; Chen, B.; Hao, H.; Wang, K. J., Optimized production of scygonadin in *Pichia pastoris* and analysis of its antimicrobial and antiviral activities. *Protein Expression & Purification* **2012,** 82, (1), 37-44. https://doi.org/10.1016/j.pep.2011.11.008
3. Xu, W. F.; Qiao, K.; Huang, S. P.; Peng, H.; Huang, W. S.; Chen, B.; Chen, F. Y.; Bo, J.; Wang, K. J., Quantitative gene expression and in situ localization of scygonadin potentially associated with reproductive immunity in tissues of male and female mud crabs, *Scylla paramamosain*. *Fish Shellfish Immun* **2011,** 31, (2), 243-251. https://doi.org/10.1016/j.fsi.2011.05.009
